# Leveraging GPT-4o for Automated Extraction and Categorization of CAD-RADS Features From Free-Text Coronary CT Angiography Reports: Diagnostic Study

**DOI:** 10.2196/70967

**Published:** 2025-09-10

**Authors:** Youmei Chen, Mengshi Dong, Jie Sun, Zhanao Meng, Yiqing Yang, Abudushalamu Muhetaier, Chao Li, Jie Qin

**Affiliations:** 1 Departments of Radiology, The Third Affiliated Hospital, Sun Yat-Sen University, 600 Tianhe Road, Guangzhou, Guangdong, 510630, China, 86 18922109279, 86 20852523108

**Keywords:** GPT-4o, radiology report, CAD-RADS, coronary computed tomography angiography (CCTA), free-text reports

## Abstract

**Background:**

Despite the Coronary Artery Reporting and Data System (CAD-RADS) providing a standardized approach, radiologists continue to favor free-text reports. This preference creates significant challenges for data extraction and analysis in longitudinal studies, potentially limiting large-scale research and quality assessment initiatives.

**Objective:**

To evaluate the ability of the generative pre-trained transformer (GPT)-4o model to convert real-world coronary computed tomography angiography (CCTA) free-text reports into structured data and automatically identify CAD-RADS categories and P categories.

**Methods:**

This retrospective study analyzed CCTA reports from January 2024 and July 2024. A subset of 25 reports was used for prompt engineering to instruct the large language models (LLMs) in extracting CAD-RADS categories, P categories, and the presence of myocardial bridges and noncalcified plaques. Reports were processed using the GPT-4o API (application programming interface) and custom Python scripts. The ground truth was established by radiologists based on the CAD-RADS 2.0 guidelines. Model performance was assessed using accuracy, sensitivity, specificity, and *F*_1_-score. Intrarater reliability was assessed using Cohen κ coefficient.

**Results:**

Among 999 patients (median age 66 y, range 58‐74; 650 males), CAD-RADS categorization showed accuracy of 0.98‐1.00 (95% CI 0.9730‐1.0000), sensitivity of 0.95‐1.00 (95% CI 0.9191‐1.0000), specificity of 0.98‐1.00 (95% CI 0.9669‐1.0000), and *F*_1_-score of 0.96‐1.00 (95% CI 0.9253‐1.0000). P categories demonstrated accuracy of 0.97‐1.00 (95% CI 0.9569‐0.9990), sensitivity from 0.90 to 1.00 (95% CI 0.8085‐1.0000), specificity from 0.97 to 1.00 (95% CI 0.9533‐1.0000), and *F*_1_-score from 0.91 to 0.99 (95% CI 0.8377‐0.9967). Myocardial bridge detection achieved an accuracy of 0.98 (95% CI 0.9680‐0.9870), and noncalcified coronary plaques detection showed an accuracy of 0.98 (95% CI 0.9680‐0.9870). Cohen κ values for all classifications exceeded 0.98.

**Conclusions:**

The GPT-4o model efficiently and accurately converts CCTA free-text reports into structured data, excelling in CAD-RADS classification, plaque burden assessment, and detection of myocardial bridges and calcified plaques.

## Introduction

The Coronary Artery Reporting and Data System (CAD-RADS), developed with support from the American College of Radiology and the American College of Cardiology, provides a standardized imaging approach for the screening, diagnosis, and treatment evaluation of coronary artery disease. CAD-RADS has been widely adopted and has had a significant impact on coronary artery disease management [1-5]. Despite the standardized approach of CAD-RADS, radiologists continue to favor free-text reports in practice. These reports often vary in style and structure, which can result in the omission of critical clinical information. This inconsistency complicates data extraction for longitudinal studies on coronary artery disease [6,7]. The issue is further exacerbated by the rapid advancements in medical technology and the increasing volume of coronary computed tomography angiography (CCTA) exams. Hospitals are now overwhelmed with unstructured reports, and manual data extraction is both time-consuming and prone to errors. Therefore, there is an urgent need for automated methods to process these reports and enhance the efficiency and accuracy of information extraction.

Traditionally, medical natural language processing (NLP) techniques have been used to extract structured data elements from medical records, particularly in the field of radiology reports [8,9]. For instance, to bridge the gap between unstructured coronary artery reports and structured data, studies have successfully used deep learning and NLP techniques to predict coronary CAD-RADS scores [10]. However, a key challenge of traditional NLP algorithms is the scarcity of high-quality annotated datasets and the high cost associated with acquiring new annotated data [11]. Even with meticulously labeled ground truth data, the relatively small size of the corpus often leads to poor model generalization or makes generalization assessments impossible. For decades, traditional artificial intelligence (AI) systems (both symbolic and neural networks) have lacked general knowledge and common-sense reasoning.

With the development of large language models (LLMs) [12,13], especially generative pre-trained transformer (GPT) models like ChatGPT, these models exhibit unique capabilities such as zero-shot and few-shot learning. Such abilities enable them to learn from prompt-based examples and perform diverse tasks [14-16]. As a result, LLMs show significant potential across multiple industries. In healthcare, LLMs, with their powerful NLP capabilities, have been used to facilitate communication between patients and clinicians [17]. In the past year, there has been a surge in the exploration of LLMs in the field of radiology reports, which has garnered increasing attention. Research has shown that LLMs can assist radiologists in tasks such as diagnosis, generating radiology reports, data extraction, error detection, incidental findings, classifying reports and data systems for various diseases, and converting unstructured free-text reports into structured formats [18-23]. Some studies have also demonstrated that LLMs excel at extracting realistic, complex, and multilingual clinical information from radiology reports, such as LI-RADS features [22,24]. However, current studies mostly rely on web-based reports and texts, with small sample sizes. Prompt engineering often involves manually created virtual reports. As a result, research on how to leverage LLMs to automatically extract structured data from vast, real-world unstructured reports and build high-quality radiology medical databases remains scarce.

This study aims to evaluate the ability of the GPT-4o model to convert real-world free-text CCTA reports into structured data and automatically identify CAD-RADS categories and Plaque Burden categories (P categories). We hypothesize that the GPT-4o model, when applied through a systematic prompt engineering approach, will demonstrate robust performance in extracting and categorizing data from CCTA reports with accuracy comparable to human experts.

## Methods

**Figure 1.**
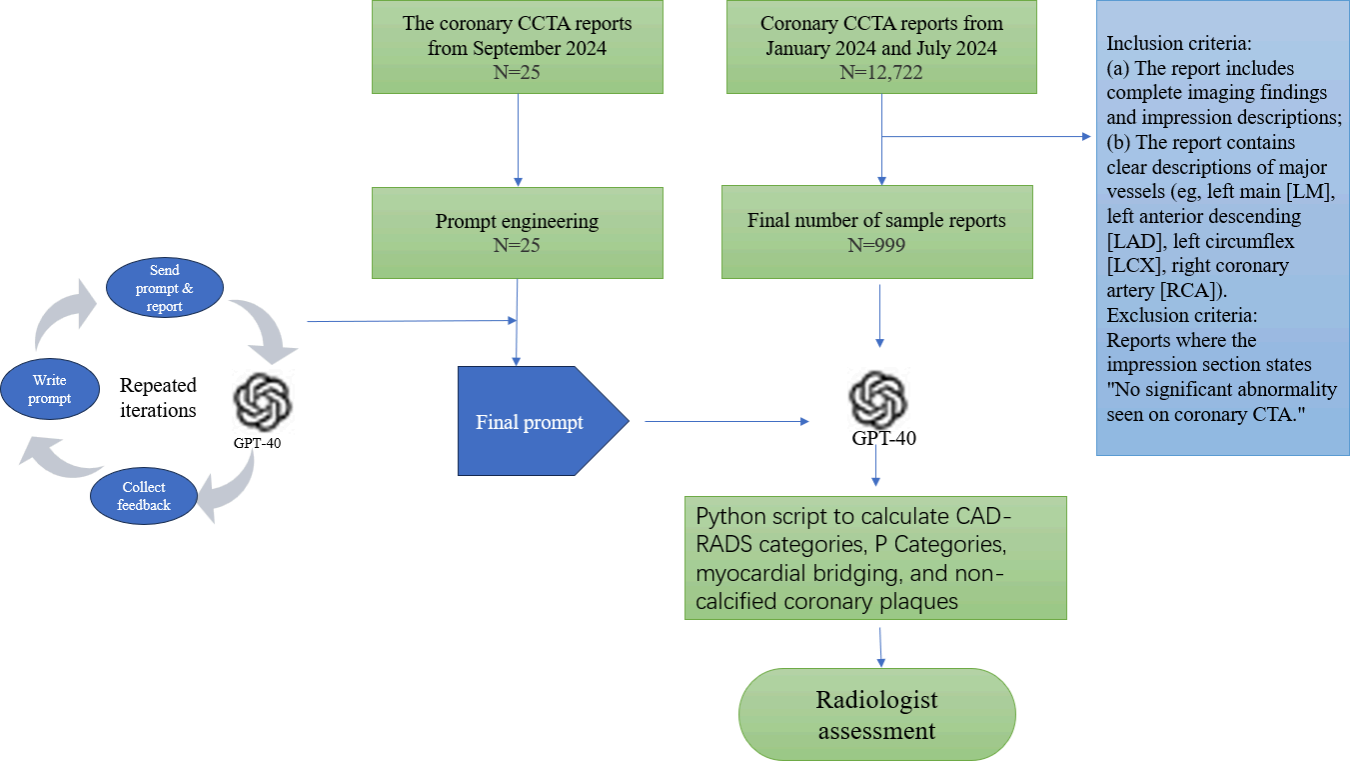
Research design flowchart. CAD-RADS: Coronary Artery Reporting and Data System; CCTA: coronary computed tomography angiography.

The overall study workflow is shown in Figure 1. The reports used for prompt engineering and the study cohort are authentic radiological reports with all sensitive information removed. This retrospective study was approved by the ethics committee of the Third Affiliated Hospital of Sun Yat-sen University (2023-042-01). All source code used in this study is publicly available in GitHub [25].

### LLM Selection

The GPT-4o model used in this study is based on the GPT-4o model released by OpenAI in May 2024 and is implemented through official API interface calls. This model is based on general domain pre-training and does not undergo institution-specific fine-tuning. The “o” in “GPT-4o” stands for “omni,” a step towards more natural human-computer interaction. It accepts any combination of text, audio, images, and video as input and generates any combination of text, audio, and image output. Compared with previous LLMs such as GPT-4, it has significant improvements on text in non-English languages and is also faster in the API and 50% cheaper. Compared with existing models, GPT-4o is particularly good at visual and auditory understanding.

### Study Sample

Reports from CCTA examinations conducted between January 2024 and July 2024 were retrieved from the Picture Archiving and Communication System (PACS) database of the Third Affiliated Hospital of Sun Yat-sen University. A total of 12,722 CCTA reports from this period were initially included. All reports were written by committee-certified radiologists and were in Chinese. Inclusion criteria were as follows: (1) reports containing complete imaging findings and impression descriptions, and (2) clear descriptions of major coronary vessels such as the left main, left anterior descending, left circumflex (LCX), and right coronary artery. Exclusion criteria involved reports where the impression stated, “no significant abnormalities detected in coronary CTA.” After applying the inclusion and exclusion criteria, 999 reports remained for analysis (sample 1). In addition, 25 CCTA reports from September 2024 (sample 2) were included for prompt engineering.

### Data Processing

The selected reports were exported to an Excel spreadsheet (Microsoft), and the author, YC, evaluated these reports, removing sensitive information such as registration numbers and names while retaining relevant details such as gender, age, imaging findings, impression descriptions, and examination dates. Except for minor typographical errors, no changes were made to the content of the reports.

### Creation of the Ground Truth

The ground truth labels for each report were established by a radiologist (YC with 3 y of experience) who evaluated all reports according to the CAD-RADS (version 2.0) guidelines [1] published in 2022, with data manually extracted into CSV format. The labeling process incorporated CAD-RADS categories, P categories, as well as the identification of myocardial bridging and noncalcified coronary plaques. In instances where YC encountered challenges in labeling, expert consultations were sought from JQ, who possesses 24 years of professional experience. To assess intrarater reliability, 200 reports were randomly selected and re-evaluated after a 15-day washout period, with agreement measured using Cohen κ coefficient.

### Prompt Engineering

The creation of prompts strictly follows the CAD-RADS 2.0 guidelines and the 18-segment coronary artery method, aiming to extract coronary artery disease-related data through simplified steps. The task of GPT-4o is to convert the free-text CCTA report into a structured format and output the data in JSON format. The initial prompt used simple instructions, such as: “Please construct the following coronary artery CTA report and output it in JSON format.” Sample 2 was used for prompt engineering, and corresponding feedback was collected. When it was found that the model output was not good or errors occurred, the instructions were modified again, such as “Please read the following report carefully, summarize it according to the CAD-RADS 2.0 guidelines, and output it in a structured form, in JSON format.” The prompt was optimized through multiple iterations until the model output the best results, and the final prompt was obtained and no longer changed. The final prompt consisted of a single step: generating structured data in JSON format based on the original report, containing the current examination date, examination technique, coronary origin, dominant vessel, and key features of each coronary artery (such as stenosis degree, plaque type, presence of myocardial bridges, presence of stents, etc.). The Chinese version of the prompt is provided in Multimedia Appendix 1. GPT-4o was instructed to generate a CSV file with process details for each report.

### Batch Feature Extraction and Classification

**Figure 2.**
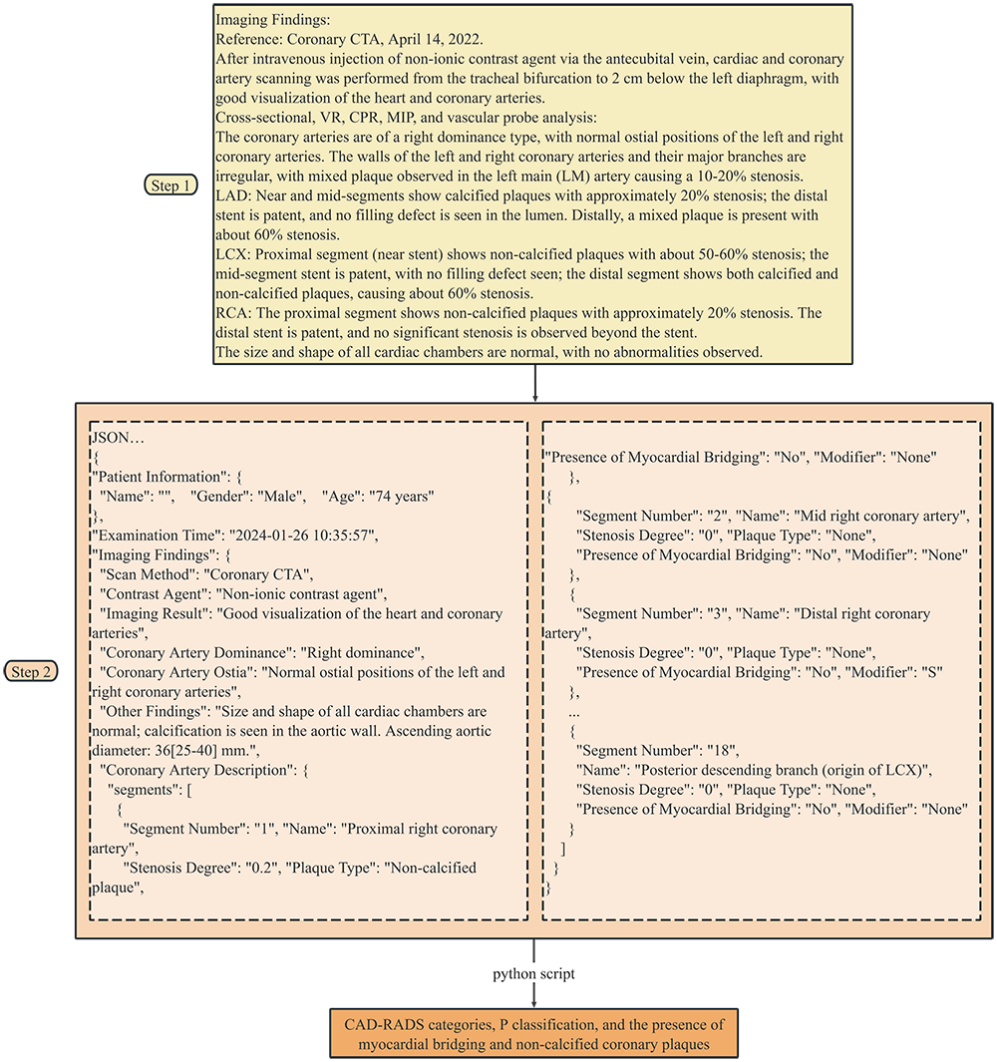
A representative example showing how GPT-4o processes and structures a coronary computed tomography angiography radiology report. The system uses a specialized prompt to transform the free-text report into a standardized JSON format, comprising 18 distinct sections (sections 1‐18, with intermediate sections abbreviated for brevity). The structured output is subsequently analyzed by a Python script to identify the Coronary Artery Reporting and Data System categories, P categories, myocardial bridge, and noncalcified plaque.

The final prompt was used to process the reports from the study cohort (Sample 1). A Python script containing the final prompt was written for batch processing of all reports. This script interacted with the GPT-4o API, sending prompts to the model one by one, with the model automatically processing the reports and converting them into CSV tables with relevant details. The characteristics of myocardial bridging and noncalcified coronary plaques were directly extracted from the model’s responses, while the CAD-RADS and P categories were programmatically determined using custom Python functions based on the CAD-RADS and P categories algorithms. An example of a report and its downstream processing is depicted in Figure 2.

The detailed calculation process of CAD-RADS and P categories algorithms can be viewed in GitHub [25].

### Error Analysis

YC conducted a review of cases with errors in the extraction of key features, specifically the CAD-RADS category and P categories. The original report, the outputs from the structured reporting process, and the final results computed using Python were jointly examined to identify the source of the errors.

### Model Performance Evaluation

The accuracy, specificity, sensitivity, and *F*_1_-score are used to evaluate the performance of the model in extracting and classifying the 4 features. The formula is as follows:


$$\text{Accuracy}=\frac{TP+TN}{TP+TN+FP+FN}$$

$$\text{Sensitivity}=\frac{TP}{TP+FN}$$

$$\text{Specificity}=\frac{TN}{TN+FP}$$

$$\text{F1}=2\times \frac{\text{Precision}\times \text{Sensitivity}}{\text{Precision}+\text{Sensitivity}},\text{Precision}=\frac{TP}{TP+FP}$$

### Statistical Analysis

In this study, baseline characteristics of the sample were described using the median and interquartile range (Q1, Q3) for continuous variables (eg, age) and frequency (n) and percentage (%) for categorical variables (eg, gender, CAD-RADS categories, P categories, and the presence of myocardial bridging and noncalcified coronary plaques). Intrarater agreement was assessed using Cohen κ statistics.

For the model-extracted features (eg, presence of myocardial bridges) and the CAD-RADS 0‐5 classification and P 0‐4 Categories, accuracy, specificity, sensitivity, and *F*_1_-score were calculated. According to the CAD-RADS 2.0 guidelines, CAD-RADS 0 indicates the absence of plaque or stenosis, and P0 is not included in the Categories. However, to evaluate the performance of the LLM in patients without plaque or stenosis, patients without plaque or stenosis were treated as P0 level in the statistical analysis.

This study was conducted using Python (version 3.8.16) as the primary programming environment and the OpenAI API (version 1.33.0) for LLM interactions. The temperature parameter mainly affects the diversity and randomness of the output of the LLM. Generally speaking, the higher the temperature, the greater the diversity and randomness of the model output, and the more unpredictable the results. To minimize model variability and ensure reproducibility, the temperature parameter was consistently set to 1E-10 across all model configurations [26,27]. Statistical analyses were performed using Python’s scikit-learn library (version 1.3.2) and SPSS statistical software (version 27; IBM Corporation).

## Results

### Study Sample

Figure 1 shows the inclusion and exclusion process for patients, while Table 1 summarizes the baseline characteristics of patients based on CAD-RADS categories and Plaque Burden categories (P categories), among other features. Initially, 12,722 coronary CTA free-text reports were screened, with 8465 reports excluded due to “no significant abnormality detected in coronary CTA,” as these patients showed no obvious coronary artery disease. An additional 3258 reports were excluded due to missing comprehensive imaging results, insufficient descriptions of findings, and the absence of clear identification of the major coronary arteries. Ultimately, 999 reports were included for analysis (median age 66 y; age range 57‐73, of whom 650 were male). All reports were successfully processed into structured data using GPT-4o, and the corresponding CAD-RADS and P categories were generated for each report.

**Table 1.** Characteristics of included patients. Unless otherwise indicated, data are numerators and data in parentheses are percentages.

Characteristics	Patients (N=999)
Gender, n (%)	
Male	650 (65.1)
Female	349 (34.9)
Age (years), median (IQR)	66 (57‐73)
CAD-RADS^a^ categories, n (%)	
0	64 (6.4)
1	236 (23.6)
2	231 (23.1)
3	224 (22.4)
4a	197 (19.7)
4b	39 (3.9)
5	8 (0.8)
Plaque Burden categories, n (%)	
0	64 (6.4)
1	310 (31.0)
2	282 (28.2)
3	294 (29.4)
4	49 (4.9)
Myocardial bridge, n (%)	438 (43.8)
Noncalcified coronary plaques, n (%)	561 (54.8)

a CAD-RADS: Coronary Artery Reporting and Data System.

### Intrarater Reliability

The κ values were 0.99 for CAD-RADS categories, 0.98 for P categories, 0.99 for myocardial bridging, and 0.98 for noncalcified plaque, indicating excellent intrarater reliability.

### Model Performance Evaluation

**Figure 3.**
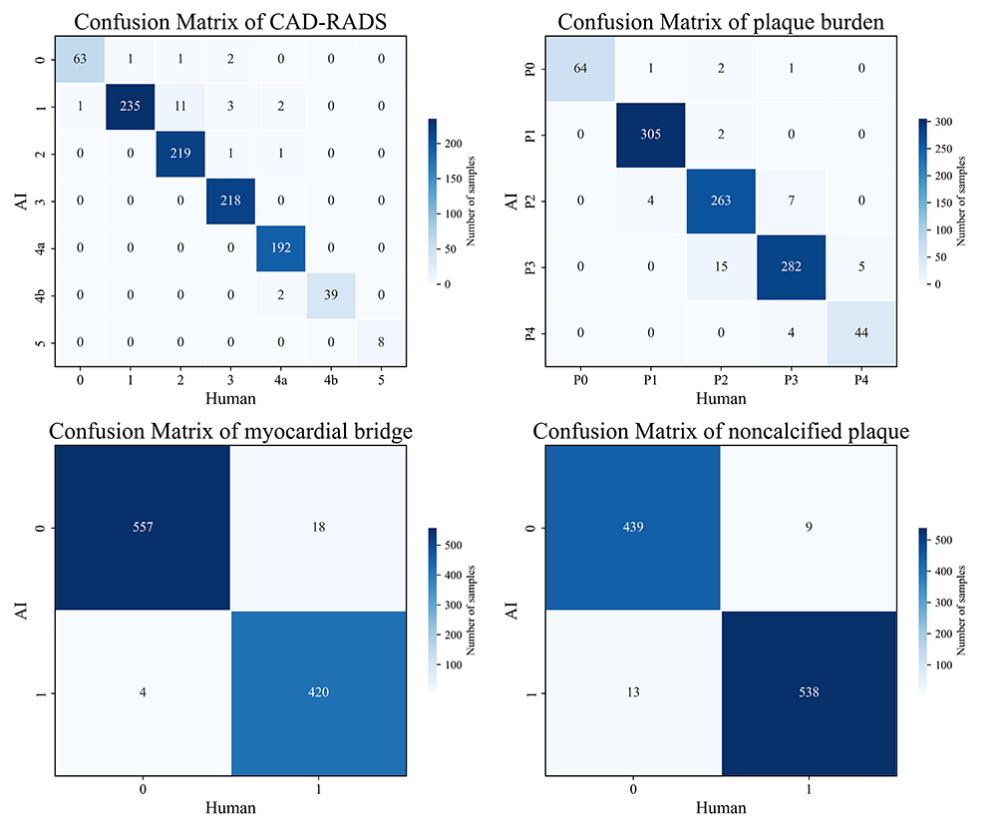
Confusion matrix of GPT-4o performance in Coronary Artery Reporting and Data System (CAD-RADS) categories and Plaque Burden categories. The confusion matrix can intuitively show the number of correct or incorrect model outputs. For example, in CAD-RADS 3 categories, the model has 218 correct answers and 6 incorrect answers.

**Figure 4.**
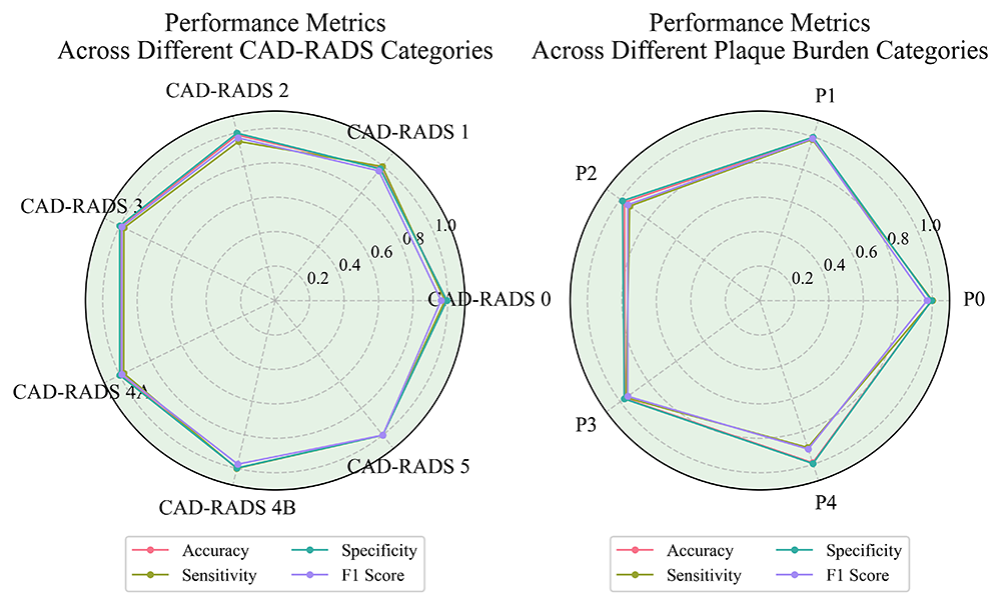
Performance metrics of generative pre-trained transformer (GPT)-4o in identifying Coronary Artery Reporting and Data System (CAD-RADS) categories and Plaque Burden categories (P categories). The radar chart can intuitively show that the model has a very high accuracy in various tasks in the CAD-RADS categories and P categories.

The confusion matrix for each CAD-RADS category, P categories, and related features from the study cohort is shown in Figure 3. The X-axis represents the number calculated by human doctors (gold standard), and the Y-axis represents the number predicted by GPT-4o. Table 2 lists the accuracy, specificity, sensitivity, and *F*_1_-score for each feature. For CAD-RADS classification (from class 0 to 5), the accuracy ranged from 0.98 to 1.00 (0.9730‐1.0000), sensitivity from 0.95 to 1.00 (0.9191‐1.0000), specificity from 0.98 to 1.00 (0.9669‐1.0000), and *F*_1_-score from 0.96 to 1.00 (95% CI 0.9253‐1.0000). For Plaque Burden P categories (P0 to P4), the accuracy ranged from 0.97 to 1.00 (95% CI 0.9569‐0.9990), sensitivity from 0.90 to 1.00 (95% CI 0.8085‐1.0000), specificity from 0.97 to 1.00 (95% CI 0.9533‐1.0000), and *F*_1_-score from 0.91 to 0.99 (95% CI 0.8377‐0.9967). For the presence of myocardial bridges in the coronary arteries, the accuracy was 0.98 (95% CI 0.9680‐0.9870), sensitivity 0.96 (95% CI 0.9376‐0.9764), specificity 0.99 (95% CI 0.9852‐0.9983), and *F*_1_-score 0.97 (95% CI 0.9630‐0.9844). For the presence of noncalcified coronary plaques, the accuracy was 0.98 (95% CI 0.9680‐0.9870), sensitivity 0.98 (95% CI 0.9710‐0.9929), specificity 0.97 (95% CI 0.9533‐0.9862), and *F*_1_-score of 0.98 (95% CI 0.9709‐0.9880). See details in Figures4  5.

**Table 2.** Performance metrics of GPT-4o in coronary CT^a^ angiography analysis.

Classifications	Accuracy (95% CI)	Sensitivity (95% CI)	Specificity (95% CI)	*F*_1_-score (95% CI)
CAD-RADS^b^ categories				
0	0.99 (0.9900‐0.9990)	0.98 (0.9492‐1.0000)	1.00 (0.9914‐0.9989)	0.96 (0.9253‐0.9916)
1	0.98 (0.9730‐0.9900)	1.00 (0.9864‐1.0000)	0.98 (0.9669‐0.9881)	0.96 (0.9450‐0.9799)
2	0.99 (0.9790‐0.9930)	0.95 (0.9191‐0.9750)	1.00 (0.9934‐1.0000)	0.97 (0.9530‐0.9845)
3	0.99 (0.9890‐0.9980)	0.97 (0.9507‐0.9912)	1.00 (1.0000‐1.0000)	0.99 (0.9747‐0.9956)
4a	0.99 (0.9900‐0.9990)	0.97 (0.9511‐0.9949)	1.00 (1.0000‐1.0000)	0.99 (0.9749‐0.9975)
4b	1.00 (0.9950‐1.0000)	1.00 (1.0000‐1.0000)	1.00 (0.9948‐1.0000)	0.97 (0.9315‐1.0000)
5	1.00 (1.0000‐1.0000)	1.00 (1.0000‐1.0000)	1.00 (1.0000‐1.0000)	1.00 (1.0000‐1.0000)
Plaque Burden categories				
0	1.00 (0.9920‐0.9990)	1.00 (1.0000‐1.0000)	1.00 (0.9914‐0.9989)	0.97 (0.9362‐0.9935)
1	0.99 (0.9870‐0.9980)	0.98 (0.9685‐0.9968)	1.00 (0.9927‐1.0000)	0.99 (0.9795‐0.9967)
2	0.97 (0.9589‐0.9800)	0.93 (0.8985‐0.9609)	0.98 (0.9754‐0.9932)	0.95 (0.9247‐0.9641)
3	0.97 (0.9569‐0.9790)	0.96 (0.9356‐0.9807)	0.97 (0.9576‐0.9831)	0.95 (0.9272‐0.9645)
4	0.99 (0.9840‐0.9970)	0.90 (0.8085‐0.9778)	1.00 (0.9916‐0.9990)	0.91 (0.8377‐0.9636)
Myocardial bridge	0.98 (0.9680‐0.9870)	0.96 (0.9376‐0.9764)	0.99 (0.9852‐0.9983)	0.99 (0.9630‐0.9844)
Noncalcified plaque	0.98 (0.9680‐0.9870)	0.98 (0.9710‐0.9929)	0.97 (0.9533‐0.9862)	0.98 (0.9709‐0.9880)

a CT: computed tomography.

b CAD-RADS: Coronary Artery Disease-Reporting and Data System.

**Figure 5.**
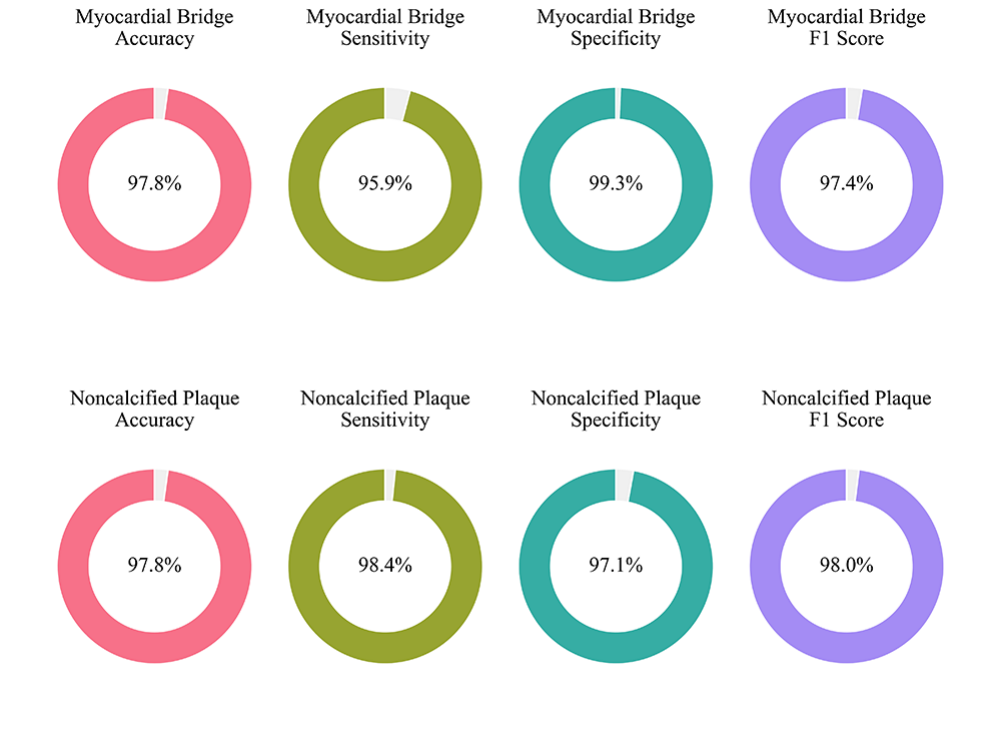
Performance metrics of generative pre-trained transformer (GPT)-4o in identifying myocardial bridge and noncalcified plaque.

### Error Analysis

This study performed an error analysis for CAD-RADS categories and P categories, involving 1998 features, with 57 errors identified, resulting in an overall error rate of 2.9%. The errors were classified into 3 main types: model output termination errors, feature extraction errors, and ambiguities in the original report descriptions, as shown in Table 3.

**Table 3.** Error analysis. In the 18-segment coronary artery model, the left circumflex is divided into proximal and distal segments with no midsegment. When “midsegment” is mentioned in the report, it is defined as the proximal segment in our system settings for the prompt engineering.

Error type	Frequency	Explanation
CAD-RADS^a^ categories		
Model output termination error	2	The model failed to output complete structured data due to unknown reasons, resulting in classification errors (n=2).
Feature extraction error	20	Although the model output the report completely, errors occurred during the feature extraction phase when converting free text to structured data, leading to classification errors (n=20). For example, in the original report, the coronary arteries had no plaques or stenosis, and the most severe stenosis was 0%. However, the model output a 10%‐20% stenosis level, misclassifying it from class 0 to class 1.
Ambiguity i then original report	0	The original report contained ambiguous or unclear descriptions, leading to erroneous structured outputs, which impacted the final classification (n=0).
Plaque Burden categories		
Model output termination error	4	The model failed to output complete structured data due to unknown reasons, resulting in classification errors (n=4).
Feature extraction error	25	Although the model output the report completely, errors occurred during the structured data conversion, particularly in recognizing coronary segment involvement, leading to classification errors (n=25). This included incorrectly identifying one or more segments as affected (n=13) or missing one or more segments (n=12), ultimately causing misclassification.
Ambiguity i then original report	6	The original report contained ambiguous or unclear descriptions, leading to erroneous structured outputs and affecting final classification (n=6). For instance, the report contained an ambiguous description of the mid-proximal segment, which was incorrectly identified as 2 separate segments during the structured output phase (n=6), resulting in misclassification.

a CAD-RADS: Coronary Artery Disease Reporting and Data System.

For CAD-RADS categories, there were 2 instances of model output termination errors, 20 instances of feature extraction errors, and no instances of ambiguities in the original report descriptions.

For overall plaque burden subclassification, there were 4 instances of model output termination errors, 25 instances of feature extraction errors, and 6 instances of ambiguities in the original report descriptions.

## Discussion

This study aimed to evaluate the capability of GPT-4o in converting unstructured coronary CTA reports into standardized formats and automatically identifying CAD-RADS categories and P categories. Our results demonstrated exceptional performance, with accuracy rates exceeding 0.97 for CAD-RADS classification and P classification. These findings suggest that LLMs could not only automate the structuring of free-text reports to enhance radiological workflow efficiency but also facilitate the extraction of valuable structured information for database construction and subsequent clinical research.

Our findings align with previous studies showing that LLMs can effectively extract structured information from medical reports. Recent research has demonstrated similar success in extracting features for various standardized reporting systems, including LI-RADS and BI-RADS [24,28]. However, our study extends beyond previous work by using a larger dataset of real-world clinical reports rather than simulated data, providing more robust evidence of GPT-4o’s clinical applicability. The model’s high performance across different classification tasks suggests its potential for broader implementation in clinical radiology workflows, particularly in standardizing reporting practices and facilitating data collection for research.

Our study further confirms LLMs' exceptional performance in extracting clinically relevant data. For instance, GPT-4 demonstrated a 96% accuracy rate in extracting lesion information from lung cancer CT reports [29], and also showed high accuracy in emergency department CT reports [30]. In our study, GPT-4o successfully identified and classified coronary artery disease, particularly in the CAD-RADS and P classification tasks, with remarkable accuracy. These findings further support LLMs' efficiency in handling complex medical tasks, especially where traditional manual extraction methods may be prone to errors due to task complexity [31].

While LLM models have shown strong capabilities in medical data extraction, hallucinations and randomness remain inherent challenges [27,32]. To reduce accuracy loss caused by hallucinations, we implemented 2 key measures. First, we set the model’s temperature to 1E-10 [27], which helps reduce variability in the model’s outputs, thus controlling randomness. Second, for the CAD-RADS and P classification tasks, we did not rely solely on the model’s output; instead, we used custom Python scripts to perform further calculations based on its structured responses. This approach ensured accuracy and validated the feasibility of our study through high performance.

Although we achieved promising results, our study still contained a small number of erroneous cases, with an overall error rate of 2.9%. Most of these errors occurred during the feature extraction phase, particularly in the CAD-RADS classification (20 out of 22 errors occurred in this phase) and P classification (25 out of 35 errors were related to segment involvement scoring). Among them, 6 errors were due to ambiguity in the original report. This error was caused by the fact that when radiologists wrote coronary CTA reports, they referred to inconsistent literature on the segmentation of coronary arteries. The descriptions of the LCX segment included the proximal LCX segment, the middle LCX segment, and the distal LCX segment. However, this study adopted the internationally accepted segmentation method of 18 coronary arteries, and the LCX only had the proximal and distal segments. For the handling of ambiguous reports, we provided the large model with relevant literature knowledge when prompt engineering and clearly informed the model that ambiguous reports generally needed to be carefully identified. After manual review and repeated iterations, the accuracy of the model for ambiguous reports was greatly improved. This shows that by giving the model specific knowledge and rules, the model can adapt to the complex clinical environment in the real world.

Due to the black-box nature of LLMs [33], we cannot directly explore the root causes of errors, but we can reasonably speculate on their sources. First, the model’s inherent hallucinations [32] may cause misleading outputs during the reasoning process of structured data. Although the temperature adjustment reduced randomness to some extent, hallucinations still influenced the reasoning process. Second, external factors such as API stability, network conditions, or computational resource limitations could also impact the model’s output. Additionally, ambiguities or inconsistencies in the original reports contributed to the occurrence of errors.

These issues highlight the need for professional oversight and intervention when using LLMs for large-scale data extraction [34] to ensure the accuracy and consistency of the extracted results. Regarding hallucinations, while we have taken measures to mitigate their impact, future research should further explore strategies to reduce hallucination phenomena, especially in clinical data extraction tasks. Furthermore, ambiguities in the original reports may arise from subtle differences in guidelines referenced by medical professionals when writing reports. To minimize this impact, radiologists should strive to adhere to internationally accepted segmentation methods, and the model should be further trained to enhance its understanding of medical knowledge, particularly the differences between various guidelines for the same disease. With these improvements, the accuracy and reliability of the model could be further enhanced.

The implementation of GPT-4o in clinical practice appears feasible given its high accuracy and efficiency. In addition, in terms of operating costs, based on OpenAI’s official pricing, the input and output of a single report in this study is approximately 2052 (SD 15) tokens, with a cost of approximately US $0.03. Batch processing of 1000 reports a day only costs US $30.80, which has the potential to be applied in clinical practice. In the RIS (Radiology Information System) of the radiology department, there is a port that can be connected to the GPT-4o API. This may require the government, hospitals, and other relevant departments to sign a patient data privacy confidentiality agreement with OpenAI. In the future, GPT-4o will have the opportunity to be applied in the radiology workflow. However, several critical considerations must be addressed before widespread adoption. Patient privacy protection remains the foremost ethical priority, requiring robust safeguards implemented in our study through a multilayered approach: beyond foundational IRB compliance, all coronary CTA reports underwent rigorous deidentification (removing Health Insurance Portability and Accountability Act–defined Protected Health Information) prior to LLM API interaction; data transmission was secured via TLS 1.2+ encryption with explicit configuration to disable third-party data retention; and access was restricted to authorized personnel through institutional role-based controls. Crucially, future clinical deployments must enforce equally stringent, auditable protocols within existing health care IT infrastructure (eg, RIS or PACS or EHR (electronic health record). Additionally, model outputs necessitate validation by qualified radiologists—particularly for high-risk cases—to ensure clinical accountability, while regular updates to the model’s knowledge base are essential to maintain alignment with evolving guidelines.

Our study has several limitations. First, all reports came from a single institution, potentially limiting the generalizability of our findings. Second, the retrospective nature of this study may have introduced selection bias, and this study used Chinese as the research subjects, mainly middle-aged and older males, so the research data may be biased, and the performance of groups in other countries may be different. Third, we only evaluated one version of GPT-4o, and performance may vary with different model versions or other LLMs. Finally, the study period was relatively short, and longer-term evaluation would be valuable for assessing the model’s consistency over time.

In conclusion, GPT-4o demonstrates remarkable capability in converting unstructured coronary CTA reports from our institution into standardized formats, suggesting significant potential for LLMs to improve radiological workflow efficiency and data standardization. If integrated with clinical decision support systems in the future, it will greatly improve the accuracy of radiologists’ diagnosis of coronary artery disease, facilitate the management of coronary artery disease, and reduce the time required to generate reports on coronary artery findings. However, the limitations outlined earlier, particularly concerning generalizability across diverse health care settings and report formats, must be rigorously addressed. Future research should prioritize multicenter validation to rigorously assess robustness and adaptability, investigate seamless integration with existing health care systems (RIS or PACS or EHR), and critically evaluate the model’s impact on downstream clinical outcomes and decision-making.

## Supplementary material

10.2196/70967 Multimedia Appendix 1 Chinese prompt words.

## Abbreviations

AI: artificial intelligence
CAD-RADS: Coronary Artery Reporting and Data System
CCTA: coronary computed tomography angiography
EHR: electronic health record
GPT: generative pre-trained transformer
LCX: left circumflex
LLM: large language model
NLP: natural language processing
P categories: Plaque Burden categories
PACS: Picture Archiving and Communication System
RIS: Radiology Information System

## References

[1] Cury RC, Leipsic J, Abbara S (2022). CAD-RADS™ 2.0 – 2022 Coronary Artery Disease – Reporting and Data System: An Expert Consensus Document of the Society of Cardiovascular Computed Tomography (SCCT), the American College of Cardiology (ACC), the American College of Radiology (ACR) and the North America Society of Cardiovascular Imaging (NASCI). Radiol Cardiothorac Imaging.

[2] Foldyna B, Szilveszter B, Scholtz JE, Banerji D, Maurovich-Horvat P, Hoffmann U (2018). CAD-RADS - a new clinical decision support tool for coronary computed tomography angiography. Eur Radiol.

[3] Basha MAA, Aly SA, Ismail AAA, Bahaaeldin HA, Shehata SM (2019). The validity and applicability of CAD-RADS in the management of patients with coronary artery disease. Insights Imaging.

[4] Xie JX, Cury RC, Leipsic J (2018). The Coronary Artery Disease-Reporting and Data System (CAD-RADS): prognostic and clinical implications associated with standardized coronary computed tomography angiography reporting. JACC Cardiovasc Imaging.

[5] Lee JW, Kim JY, Han K (2021). Coronary CT angiography CAD-RADS versus coronary artery calcium score in patients with acute chest pain. Radiology.

[6] Bosmans JML, Weyler JJ, De Schepper AM, Parizel PM (2011). The radiology report as seen by radiologists and referring clinicians: results of the COVER and ROVER surveys. Radiology.

[7] Wallis A, McCoubrie P (2011). The radiology report--are we getting the message across?. Clin Radiol.

[8] Hao T, Huang Z, Liang L, Weng H, Tang B (2021). Health natural language processing: methodology development and applications. JMIR Med Inform.

[9] Pathak J, Kho AN, Denny JC (2013). Electronic health records-driven phenotyping: challenges, recent advances, and perspectives. J Am Med Inform Assoc.

[10] Tariq A, Van Assen M, De Cecco CN, Banerjee I (2022). Bridging the gap between structured and free-form radiology reporting: a case-study on coronary CT angiography. ACM Trans Comput Healthcare.

[11] Wu H, Wang M, Wu J (2022). A survey on clinical natural language processing in the United Kingdom from 2007 to 2022. NPJ Digit Med.

[12] Touvron H, Lavril T, Izacard G (2023). Open and efficient foundation language models. arXiv.

[13] Achiam J, Adler S, Agarwal S (2023). Gpt-4 technical report. arXiv.

[14] Lyu Q, Tan J, Zapadka ME (2023). Translating radiology reports into plain language using ChatGPT and GPT-4 with prompt learning: results, limitations, and potential. Vis Comput Ind Biomed Art.

[15] Sun Y, Zheng Y, Hao C, Qiu H (2021). NSP-BERT: a prompt-based few-shot learner through an original pre-training task--next sentence prediction. arXiv.

[16] Wei J, Bosma M, Zhao VY (2021). Finetuned language models are zero-shot learners. arXiv.

[17] Lai X, Chen J, Lai Y (2025). Using large language models to enhance exercise recommendations and physical activity in clinical and healthy populations: scoping review. JMIR Med Inform.

[18] Adams LC, Truhn D, Busch F (2023). Leveraging GPT-4 for post hoc transformation of free-text radiology reports into structured reporting: a multilingual feasibility study. Radiology.

[19] Nakaura T, Yoshida N, Kobayashi N (2024). Preliminary assessment of automated radiology report generation with generative pre-trained transformers: comparing results to radiologist-generated reports. Jpn J Radiol.

[20] Biswas S, Khan S, Awal SS (2024). Can ChatGPT write radiology reports?. Chin J Acad Radiol.

[21] Cao JJ, Kwon DH, Ghaziani TT (2023). Accuracy of information provided by ChatGPT regarding liver cancer surveillance and diagnosis. AJR Am J Roentgenol.

[22] Sievert M, Conrad O, Mueller SK (2024). Risk stratification of thyroid nodules: assessing the suitability of ChatGPT for text-based analysis. Am J Otolaryngol.

[23] Shan G, Chen X, Wang C (2025). Comparing diagnostic accuracy of clinical professionals and large language models: systematic review and meta-analysis. JMIR Med Inform.

[24] Gu K, Lee JH, Shin J (2024). Using GPT-4 for LI-RADS feature extraction and categorization with multilingual free-text reports. Liver Int.

[25] Lichao312214129/code_for_cad-RADS. GitHub.

[26] Bhayana R, Nanda B, Dehkharghanian T (2024). Large language models for automated synoptic reports and resectability categorization in pancreatic cancer. Radiology.

[27] Holtzman A, Buys J, Du L, Forbes M, Choi Y (2019). The curious case of neural text degeneration. arXiv.

[28] Cozzi A, Pinker K, Hidber A (2024). BI-RADS category assignments by GPT-3.5, GPT-4, and Google Bard: a multilanguage study. Radiology.

[29] Fink MA, Bischoff A, Fink CA (2023). Potential of ChatGPT and GPT-4 for data mining of free-text CT reports on lung cancer. Radiology.

[30] Infante A, Gaudino S, Orsini F (2024). Large language models (LLMs) in the evaluation of emergency radiology reports: performance of ChatGPT-4, perplexity, and Bard. Clin Radiol.

[31] Abu-Ashour w, Emil S, Poenaru D (2024). Using artificial intelligence to label free-text operative and ultrasound reports for grading pediatric appendicitis. J Pediatr Surg.

[32] Ji Z, Lee N, Frieske R (2023). Survey of hallucination in natural language generation. ACM Comput Surv.

[33] Ehsan U, Riedl M Explainable AI reloaded: challenging the XAI status quo in the era of large language models.

[34] Parillo M, Vaccarino F, Beomonte Zobel B, Mallio CA (2024). ChatGPT and radiology report: potential applications and limitations. Radiol Med.

